# Memory Decline in Down Syndrome and Its Relationship to iPF2alpha, a Urinary Marker of Oxidative Stress

**DOI:** 10.1371/journal.pone.0097709

**Published:** 2014-06-05

**Authors:** Panagiotis Zis, Patrick McHugh, Andrew McQuillin, Domenico Praticò, Mark Dickinson, Sima Shende, Zuzana Walker, Andre Strydom

**Affiliations:** 1 University College London, Division of Psychiatry, London, United Kingdom; 2 Division of Pharmacy and Pharmaceutical Sciences, School of Applied Sciences University of Huddersfield, Queensgate, Huddersfield, United Kingdom; 3 University College London, Molecular Psychiatry Laboratory, London, United Kingdom; 4 Department of Pharmacology and Center for Translational Medicine, Temple University, Philadelphia, Pennsylvania, United States of America; 5 Enfield Integrated Learning Disability Team, Enfield, United Kingdom; Louisiana State University Health Sciences Center, United States of America

## Abstract

**Background:**

Lipid peroxidation may be a marker of free-radical-mediated injury associated with Alzheimer’s disease (AD). We aimed to investigate whether changes in lipid peroxidation is associated with cognitive decline in individuals with Down syndrome over a 4-year period.

**Methods:**

Thirty-two adults with DS participated in a longitudinal study with urinary isoprostane 8,12-iso-iPF2alpha (iPF2alpha) assays at baseline and four years follow-up. Informants rated their functional ability and memory function and the adults with DS attempted assessments of language skills and memory. Twenty-six individuals completed assessments of memory (Modified Memory Object Task, MOMT), adaptive behavior (ABAS), and receptive vocabulary (British Picture vocabulary, BPVS) at both time-points.

**Results:**

Overall change in iPF2alpha level was negatively correlated with change in the MOMT score (Spearman’s Rho = −0.576, p = 0.006), i.e., increased lipid peroxidation was correlated with worse memory functioning over time. An increase of ≥0.02 ng/mg creatinine iPF2α had good sensitivity (85.7%), positive predictive value (75%,), specificity (85.7%) and negative predictive value (92.3%) for memory decline.

**Conclusion:**

Change in iPF2alpha over time may have potential as a biomarker for memory decline in Down syndrome and potentially also help to track progression of MCI to AD in the general population.

## Introduction

Oxidative stress reflects the biological imbalance between oxidant production and the ability to detoxify the system of reactive oxygen species (ROS) and repair the damage they cause. Although low levels of ROS are physiologically important, high concentrations above the clearance capacity cause mitochondrial dysfunction, cellular damage, and cell death [1], implicating oxidative stress as an important mechanism of age-related pathology [2] such as the cognitive decline associated with Alzheimer’s Disease (AD) [3].

Down syndrome (DS) has been associated with disturbed oxidative metabolism, which is assumed to be due to a variety of mechanisms [4], and most adults with DS will eventually show the clinical or neuropathological hallmarks of AD [5]. Although this may be in large part due to the triplication of the APP gene in DS, increasing evidence suggests that oxidative stress and inflammation may also play a role in AD pathogenesis associated with the syndrome [6].

Lipid peroxidation is one of the major outcomes of oxidative stress following free-radical-mediated injury, leading to the generation of various end products [7]. Isoprostane 8,12-iso-iPF2alpha (iPF2alpha) are isomers of the prostagladin F2alpha produced by ROS attack on polyunsaturated fatty acids. They are chemically stable, sensitive and specific biomarkers of lipid peroxidation in vivo [8] and have been shown to be increased in Alzheimer’s disease, and may mediate the neuronal response to oxidative stress [9]. Elevated levels of iPF2alpha have been demonstrated in living adults in DS [10].

Given the DS population’s much increased risk for dementia, they are an important population in which to study biomarkers of AD. We aimed to investigate (in an existing research cohort of adults with DS) whether a peripheral marker of oxidative stress, specifically a change over time in lipid peroxidation (measured with urinary iPF2alpha) is associated with cognitive decline in people with DS, and whether this is related to anti-oxidative enzyme levels, including Cu/Zn Superoxide dismutase (SOD1).

## Materials and Methods

### Study Population, iPF2alpha, Enzyme Assays and Psychometric Measurements

We used a within-syndrome design to examine the association between urinary markers of oxidative stress and changes in cognitive functioning over time in adults with DS. We have previously established a cohort of 32 individuals with DS who underwent cognitive testing and donated urine samples for isoprostane 8,12-iso-iPF2alpha (iPF2alpha) analysis and blood samples for Cu/Zn superoxide dismutase (SOD1), overall superoxide dismutase activity (SOD) and glutathione peroxidase (GPx) analysis. Assays for SOD1, SOD, GPx were measured at baseline, using methods described elsewhere [11], [12].

Urine iPF2alpha levels (corrected for creatinine concentrations) were measured at both baseline and at follow-up four years later (mean length of follow up 55.46 months, range 46 to 61 months). Collection of and processing of urine samples were similar at both time points. At baseline, urine ipf2alpha levels were measured using methods described elsewhere [11]. At follow-up, urine samples were hydrolysed with a 1/10 volume of 1N HCL and diluted 1∶4 with PBS prior to analysis. All reagents for subsequent 8-iso-PGF2alpha analysis were obtained from Cell Biolabs, Inc. 8-iso-PGF2alpha standards ranging from 200 µg/mL to 0.2 µg/mL were used to generate a standard curve. Urine samples or standards were mixed with a 8-iso-PGF2alpha-HRP conjugate for 1 hour at room temperature in a 96-well plate. Wells were washed 5 times with 300 µL wash buffer (1X PBS, 0.05% Tween-20). One hundred microlitres of HRP substrate was added to each well and mixed for 30 mins at room temp. The enzyme reaction was stopped with 100 µl 1 M phosphoric acid the enzyme reaction by adding 100 µL of Stop Solution to each well. The absorbance was determined for each well at 450 nM (POLARstar OPTIMA plate-reader, BMG) and quantified by comparing with the known predetermined standard curve.

To determine creatinine levels, urine samples were diluted 1∶20 with Milli-Q water. Creatinine standards ranging from 20 mg/dl to 0.078 mg/dl were used to generate a standard curve. Fifty microliters of standards and samples were added to a 96-well plate with 200 µl creatinine reaction reagent (Cell Biolabs, Inc.) and incubated for 30 mins with shaking at room temp. Initial absorbance values were determined for each well at 490 nM (POLARstar OPTIMA plate-reader). Fifty microliters creatinine quencher (Cell Biolabs, Inc.) was added to each well, mixed thoroughly and incubated for 10 mins at room temp. A final absorbance value was determined at 490 nM and subtracted from the initial absorbance giving a corrected absorbance value. Samples were quantified using by comparing the corrected absorbance against the known predetermined standard curve.

Details of the psychometric assessments and population characteristics have also been described elsewhere [11], [12]. In brief, informants (who were in most cases family members) completed a measure of adaptive behavior (Adaptive Behaviour Assessment Scale; ABAS [13]) and a dementia screen (the cognitive scale of the Dementia Scale for people with Learning Disabilities; DLD [14]). Participants who were able to, completed a measure of receptive vocabulary (British picture vocabulary scale; BPVS II [15]), and a modification of an object memory task (MOMT) based on the Fuld object memory test [16], [17]. We modified the task by reducing the number of objects to 6, and the number of trials to two.

### Ethics Statement and Consent Procedures

The study was approved by the National NHS research ethics committee and we followed the Mental Capacity Act, UK (2005) if participants did not have the capacity to consent for themselves, by gaining agreement from carers. Written informed consent was obtained for all participants.

### Data Analyses

Data was analysed with Statistical Package for the Social Sciences (SPSS) version 14 [18]. The SOD/GPx activity ration was calculated from the logarithms of the activities because of the different orders of magnitude [19]. Correlations between urinary iPF2alpha levels and baseline factors were examined using Spearman’s correlations. Correlations between the changes in psychometric and functional assessments scores (BPVS, ABAS, MOMT) were examined using Spearman’s correlations. Mann Whitney U tests were used to compare scores between groups. The significance level was set at 0.05.

## Results

### Demographics and Psychometric Tests

Twenty-six out of 32 individuals with DS completed psychometric testing at both time points. Two of the original participants refused to participate again, and four were lost to follow-up. We have previously described the demographic features of this cohort [12]. In summary, the 26 participants had a mean age of 36.65±7.05 years, and half of them where males. None were diagnosed with Alzheimer’s disease at baseline or during the follow-up period.

Table 1 summarizes the average scores of all psychometric tests and subtests at follow up and shows the mean change of each score between baseline and follow-up.

**Table 1:** Mean scores and 95% confidence intervals of psychometric tests at baseline, follow up and change between time points.

	Baseline (t1)	Follow-up (t2)	Mean change i.e. t2–t1
**ABAS**			
Functional academics	30.4 (21.6–39.2)	25.1 (17.9–32.3)	–5.3 (−9.3–−1.4)
Total score	366.8 (312.7–421.0)	317.9 (263.9–371.8)	–49.0 (−71.1–−26.9)
**BPVS**			
Raw score	61.6 (53.1–70.2)	55.7 (46.3–65.1)	–6.0 (−9.1–−2.8)
Age equivalent, in months	73.1 (62.3–83.9)	66.4 (54.4–78.4)	n/a
**MOMT**			
Delayed recall	4.7 (4.3–5.2)	4.8 (4.4–5.3)	0.1 (−0.4–0.6)
Total score	12.6 (11.9–13.4)	13.0 (12.3–13.7)	0.4 (−0.4–1.2)

Although total MOMT scores remained relatively stable, 7(33.3%) participants showed decline on MOMT scores between T1 and T2.

### iPF2alpha Measurements and Enzyme Assays

Data on urinary iPF2α were available for 24 patients at baseline and follow-up. For these 24 patients, at baseline, the range of iPF2alpha was 0.16 to 3.18 ng/mg creatinine (mean 1.39±0.79) and at follow-up 0.52 to 5.27 ng/mg creatinine (mean 1.38±1.23). The change between iPF2α levels at follow up and at baseline ranged from −2.60 to +4.30 ng/mg creatinine (mean 0.003±1.56). Table 2 presents individual values for all patients, at both baseline and follow-up.

**Table 2:** Individual values of urinary ipf2alpha for both baseline and follow up.

Patient	Gender	Age at baseline,in years	Ipf2a at baseline,in ng/mg creatinine	Ipf2a at follow up,in ng/mg creatinine
#1	Female	40	.22	.89
#2	Female	22	1.19	.87
#3	Female	33	1.75	.87
#4	Male	45	1.04	4.42
#5	Female	27	1.22	.57
#6	Female	30	.86	1.16
#7	Male	26	3.18	.58
#8	Male	31	1.45	1.16
#9	Female	36	1.31	.62
#10	Male	35	2.12	1.79
#11	Female	30	1.05	1.04
#12	Female	25	1.24	3.49
#13	Male	32	.97	5.27
#14	Female	22	1.75	.67
#15	Male	36	1.12	.90
#16	Male	39	2.86	1.41
#17	Female	35	3.16	1.06
#18	Female	40	1.33	.68
#19	Male	35	.72	.97
#20	Male	27	1.51	.52
#21	Female	24	.16	1.44
#22	Female	30	1.55	.62
#23	Male	18	.60	1.27
#24	Male	41	.93	.95

The mean change in the iPF2α levels did not correlate with age (Spearman’s rho = 0.074, p = 0.731), nor did it differ between males and females (Student’s t-test, t value = −0.715, p = 0.482), patients with thyroid disorder and those without (Mann-Whitney U, Z value = −0.400, p = 0.689), mild ID or moderate to severe ID (Mann-Whitney U, Z value = −0.306, p = 0.760), and white UK or those of other ethnicity (Mann-Whitney U, Z value = −1.333, p = 0.182). However, those receiving vitamins and supplements at baseline presented with increased urinary levels of iPF2α (mean change +0.882 ng/mg) compared to those who were not (mean change −0.404 ng/mg); t value = −1.975; p = 0.02.

### Relationship between Oxidative Enzymes and Urinary iPF2alpha

Table 3 summarizes the correlations between baseline enzyme assays, urine iPF2alpha at follow-up, and change in iPF2alpha over time. Correlations between baseline enzyme assays and baseline urine iPF2alpha have been presented elsewhere [11]. Enzyme assays at baseline were not correlated significantly with urinary iPF2alpha at follow up, or the change in urinary iPF2alpha levels over the 4-year period. However, there was a trend towards a negative relationship between overall SOD function at baseline and change in iPF2alpha over time. A trend towards a similar relationship was also noted for GPx (i.e. better SOD function and higher GPx levels may be related to less change in iPF2alpha over 4 years).

**Table 3:** Correlations between anti-oxidant enzymes in blood and urinary iPF2alpha.

		iPF2alpha at follow-up (t2)	Change in iPF2alpha (t2–t1)
SOD function	Spearman’s Rho	−0.406	−0.444
	p	0.076	0.065
	N	20	18
SOD enzyme units	Spearman’s Rho	−0.128	−0.065
	p	0.612	0.811
	N	18	16
GPx	Spearman’s Rho	−0.400	−0.420
	p	0.081	0.082
	N	20	18
SOD function/GPx	Spearman’s Rho	0.186	0.207
	p	0.431	0.409
	N	20	18

### Relationship between Urinary iPF2alpha and Cognitive Ability Changes

Table 4 lists the correlations between levels of iPF2alpha and performance over 4 years on tests of functional ability, memory or language ability. Correlations between baseline psychometric measures and baseline urine iPF2alpha have been presented elsewhere [11]. There were no statistically significant correlations between levels of ipF2alpha level at follow up or its change over 4 years and ABAS total score, or with ABAS functional academic score. However, overall change in iPF2alpha level was negatively correlated with change in the MOMT total score (Spearman’s Rho = −0.516, p = 0.017) and MOMT delayed recall score (Spearman’s Rho = −0.576, p = 0.006). Increases in lipid peroxidation were correlated with worse memory functioning over time. An increase equal to or more than 0.02 ng/mg creatinine in iPF2alpha identified 6 out of 7 participants who showed declines on memory tasks (sensitivity = 85.7%; positive predictive value = 75%) and included only 2 of 14 participants who did not show memory decline (specificity = 85.7%; negative predictive value = 92.3%).

**Table 4:** Correlations between changes in psychometric measures (scores at follow up minus score at baseline), and urinary iPF2alpha titers.

		iPF2alpha at follow-up	Change in iPF2alpha
Change in ABAS total score	Spearman’s Rho	0.215	0.188
	p	0.292	0.379
	N	26	24
Change in ABAS functional academic score	Spearman’s Rho	0.118	−0.241
	p	0.566	0.256
	N	26	24
Change in BPVS raw score	Spearman’s Rho	−0.204	−0.178
	p	0.363	0.440
	N	22	21
Change in MOMT total score	Spearman’s Rho	−0.195	−0.516
	p	0.397	0.017*
	N	21	21
Change in MOMT delayed recall score	Spearman’s Rho	−0.258	−0.576
	p	0.259	0.006**
	N	21	21

*means p<0.05 when **means p<0.01.

## Discussion

### Findings

We have previously shown that serum levels of SOD predicts memory decline over time [12]. In the present study, we have explored the relationship between cognitive change over time in adults with DS and urinary markers of oxidative stress, which suggests that there is a correlation between increased oxidative stress measured with lipid peroxidation and memory decline in adults with DS over time. Our results suggest that an increase in iPF2alpha, but not absolute iPF2alpha titers, is an early marker of progression to AD in DS, and that increased lipid peroxidation can predict decline on memory tests in this population with good sensitivity and specificity.

### Implications

Our results suggest that increases in urinary iPF2alpha over time are associated with the memory decline that is typical of AD in adult participants with DS, an ultra-high risk group for developing dementia.

In keeping with our results, a general population study of AD found that an increase in isoprostane levels in CSF (rather than absolute values) was associated with progression of mild cognitive impairment (MCI) to AD and with cognitive decline over time, and that this marker was more predictive of progression than other AD biomarkers [20]. Taken together, our study (in adults with DS) and the CSF study (with participants without DS) suggest that markers of oxidative stress have considerable potential as a biomarkers of the cognitive decline suggestive of MCI, and of progression of AD symptoms. Although absolute values of isoprostanes may not have much value in screening for AD, increased levels over time have considerable potential to track progression of disease. Given that urine samples are easier to obtain than CSF, our results have important practical implications.

It has been suggested that oxidative stress is an important consequence of the specific biology of DS, and is likely to play a role in some of the co-morbidities associated with DS, including the tendency to develop AD [4]. Oxidative damage may lead to enhanced amyloid beta peptide (Aβ) production [21]–[24] and in a recent study, Cenini et al. showed that soluble and insoluble Aβ and oligomers increase as a function of age in DS frontal cortex, and that Aβ40 correlated with protein carbonyls (an oxidative stress marker), which suggests that oxidative damage may contribute to the onset and progression of AD pathogenesis in DS [25]. The exact mechanism is still unknown, but Yang et al. showed that ROS enhanced amyloid toxicity in the neurons of APP/PS1 mice [26], an AD mouse model with excessive amyloid production which is also found in DS, while Di Domenico et al demonstrated that oxidation of proteins is an early event in DS and might contribute to neurodegenerative phenomena [27].

Peripheral biomarkers of oxidative stress in Down syndrome include SOD [12], [26], [28], GPx [6], [29], uric acid [29], plasma melatonin, urinary kynurenine [30], gelsolin [31], nitric oxide [32], [33] and neopterin [34]. However, oxidative stress or lipid peroxidation may not necessarily be increased in DS. Tolun et al. compared the levels of urinary allantoin and F2-isoprostanes between individuals with DS and controls without finding any significant difference [35]. Moreover, Campos et al assessed a set of urinary oxidative stress biomarkers in children with DS including 8-hydroxy-2′-deoxyguanosine (8-OHdG), isoprostane 15-F2t-IsoP, thiobarbituric acid-reacting substances (TBARS), advanced glycation end products (AGEs), dityrosine (diTyr), hydrogen peroxide (H2O2) and nitrite/nitrate (NOx) and found that only levels of diTyr were increased in DS, although no differences were obtained when hypothyroid DS children were excluded [36]. In another study, Campos et al, aimed to assess the same set of oxidative and nitrosative stress biomarkers in urine samples of adolescents and adults with DS, with and without hypothyroidism and concluded that some biomarkers such as 8-OHdG, 15-F2t-IsoP and TBARS may be prone to age effects, renal function, and thyroid function [37]. In contrast, we did not find any correlation between changes in iPF2alpha over time and age, nor an effect for thyroid disorder.

It has been demonstrated that premature aging in kidneys of DS patients could lead to an impaired renal function [38]. For this reason, not only the oxidative status but also the alteration of the glomerular filtration rate could affect nitrite and nitrate levels in DS patients. Therefore, Ripoll et al conducted a comparative study assessing nitrosative stress biomarkers in plasma samples from adult DS patients [39], concluding that the increased levels of nitrates found in urine samples of adult DS patients [37] are also found in plasma samples, which confirms the use of nitrogen reactive species as stress biomarkers in adult DS patients.

Increases in the anti-oxidant systems in DS (such as SOD) help to protect against increased oxidative stress [40]. Our results are in keeping with these studies – we have previously demonstrated a positive relationship between SOD1 and better memory function, while the present results indicate that when the protective mechanisms fail over time and lipid peroxidation increases, it is associated with decline in memory function.

Lastly, increased lipid peroxidation has been shown to precede amyloid plaque formation in some studies of animal models [24] and lipid peroxidation may trigger neurodegeneration [22]; reduction of oxidative stress could therefore be a potential treatment target in the DS population.

### Strengths and Limitations

We undertook a prospective cohort study of adults with DS, in which we managed to follow up 80% of the initial study group over an unusually long period (4 years). We have included participants with the full range of cognitive abilities associated with DS, and our sample is therefore representative of the wide variation in intellectual phenotype. However, our sample size was relatively small, and our results need to be confirmed with a replication study.

We were not able to control for all possible confounders as, for example, cognitive performance may be influenced by educational exposure, though in the UK all adults with DS have equal access to education. Moreover, it is likely that dietary intake of food with antioxidant properties varied across participants and we did not collect sufficient detail on compliance with vitamin supplementation during the follow-up period to include this in our analysis.

The technique of measurement of ipf2alpha at follow up was different at follow-up compared to baseline (where gas chromatography and mass spectrometry i.e. GC/MS was used). When immunoassays are performed under appropriate conditions, values obtained for isoprostanes obtained by immunoassay often correlate well (r^2^>0.8) with values obtained by the standard GC/MS method [41].

Among the followed-up population, there were no patients with translocation, and there was only one patient with mosaicism. Therefore, we were not able to study the relationship between iPF2alpha levels and these categories in more detail. The study was powered to detect correlations of moderate strength and it is therefore possible to have missed more subtle relationships. For example, a significant relationship between SOD and GPx and changes in lipid peroxidation may become apparent in larger studies.

### Conclusion

For the first time, it is suggested that change in urinary iPF2alpha over time is associated with memory decline in people with DS, suggesting that sequential measurements of urinary iPF2alpha may have potential as biomarker for cognitive decline and progression to AD in this population. This preliminary finding adds to similar studies in the general population, which showed increased isoprostanes in the CSF of people with MCI who have progressed to full-blown AD. Furthermore, the association between an increase in lipid peroxidation and cognitive decline in relatively young adults with DS suggests that reduction of oxidative stress and consequent lipid peroxidation may be a potential treatment strategy to reduce the high risk for AD in this population.
